# Supplementary material for: Contribution of Structure Learning Algorithms in Social Epidemiology: Application to Real-World Data
Source: Int J Environ Res Public Health. 2025 Feb 27;22(3):348. doi: 10.3390/ijerph22030348 (PMC11941975; doi:10.3390/ijerph22030348)
Supplement: Supplementary file 1 [file ijerph-22-00348-s001.zip › ijerph-3451922-supplementary.pdf]

Description of the cohort.

Table S1: Description of the population of the cohort (2010).

	SIRS cohort	Total population[1]
	N (%)	N (%)
**Age (in years)**		
18-29	374 (12.4%)	1,180,614 (22.3%)
30-44	875 (29.1%)	1,552,369 (29.3%)
45-59	837 (27.8%)	1,271,540 (24.0%)
60-74	619 (20.6%)	795,665 (15.0%)
75+	301 (10.0%)	489,085 (9.25%)
**Gender**		
Men	1,187 (39.5%)	2,488,082 (47.0%)
Women	1,819 (60.5%)	2,801,188 (53.0%)
**Origin**		
French, French parents	2,002 (66.6%)	
French, foreign parents	610 (20.3%)	
Migrant	394 (13.1%)	
**Education level**		
Primary or none	316 (10.5%)	635,736 (12.0%)
Secondary	1,247 (41.5%)	2,483,682 (47.0%)
Tertiary	1,443 (48.0%)	2,169,857 (41.0%)
**Employment status**		
Employed	1,596 (53.1%)	3,075,468 (58.1%)
Unemployed	212 (7.1%)	418,256 (7.9%)
Inactive	1,198 (39.9%)	1,795,550 (33.9%)
**Income**		
1st quintile	687 (22.9%)	
2nd quintile	637 (21.2%)	
3rd quintile	645 (21.5%)	
4th quintile	481 (16.0%)	
5th quintile	556 (18.5%)	
**Health insurance status**		
None or SHI only	347 (11.5%)	
SHI and VHI	2,659 (88.5%)	
**Health relatives**		
No	1,866 (62.1%)	
Yes	1,140 (37.9%)	
**Social integration**		
1st quartile	754 (25.1%)	
2nd quartile	753 (25.0%)	
3rd quartile	752 (25.0%)	
4th quartile	747 (24.9%)	
**Chronic Disease**		
No	1,854 (61.7%)	
Yes	1,152 (38.3%)	
**Perceived health status**		
Good	2,190 (72.9%)	
Bad-Average	816 (27.1%)	
**Indirect access to care**		
No	1,613 (53.7%)	
Yes	1,393 (46.3%)	
**Direct access to care**		
No	324 (10.8%)	
Yes	2,682 (89.2%)	

1: Census data

Indirect access to care.

Table S2. Results of the non-automated approach with "indirect access to care" (IAC) as outcome.

	Link to DAC[1]	Direction[2]	Strength[3]
**Age**	Yes	To IAC	
18-29			Ref
30-44			0.95 (0.72 to 1.26)
45-59			1.40 (1.05 to 1.87)
60-74			2.31 (1.70 to 3.15)
75+			2.52 (1.75 to 3.64)
**Gender**	Yes	To IAC	
Men			Ref
Women			1.34 (1.14 to 1.58)
**Origin**	No	-	
French, French parents			-
French, foreign parents			-
Migrant			
**Education level**	Yes	To IAC	
Primary or none			Ref
Secondary			1.18 (0.89 to 1.57)
Tertiary			1.64 (1.20 to 2.25)
**Employment status**	No	-	
Employed			-
Unemployed			-
Inactive			-
**Income**	Yes	To IAC	
1st quintile			Ref
2nd quintile			1.03 (0.80 to 1.31)
3rd quintile			1.18 (0.92 to 1.52)
4th quintile			1.70 (1.29 to 2.45)
5th quintile			1.66 (1.24 to 2.22)
**Health insurance status**	Yes	To IAC	
None or SHI only			Ref
SHI and VHI			1.67 (1.25 to 1.33)
**Health relatives**	Yes	To IAC	
No			Ref
Yes			1.22 (1.03 to 1.44)
**Social integration**	No	-	
1st quartile			-
2nd quartile			-
3rd quartile			-
4th quartile			-
**Chronic Disease**	Yes	To IAC	
No			Ref
Yes			2.64 (2.22 to 3.13)
**Perceived health status**	Yes	To IAC	
Good			Ref
Bad-Average			1.93 (1.59 to 2.35)

1: significant association found in final logistic regression modelling IAC by other variables identified as its potential direct determinants by experts; 2: based on the initial network defined by expert; 3: given here by the Odds Ratio and its 95% confidence interval as estimated by the final logistic regression.

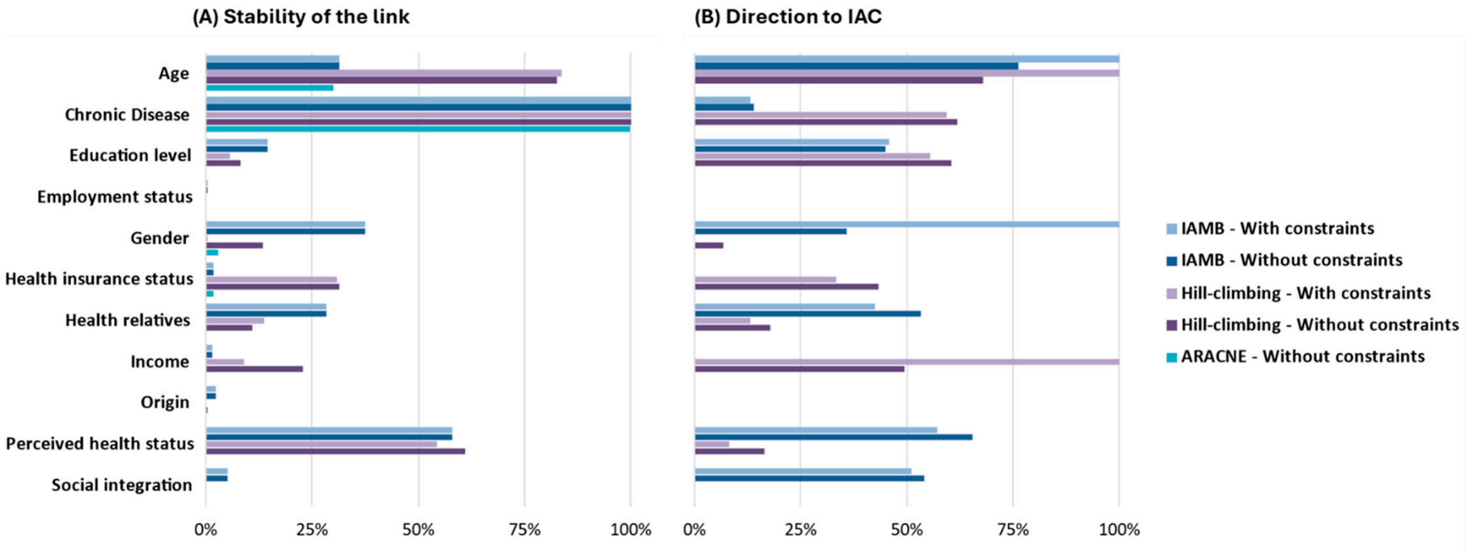

Figure S1. Results of structure learning approach with "indirect access to care" (IAC) as outcome. (A)*: Strength/stability is the relative apparition of the link among the bootstrap replicates; (B) frequency of the direction of the link to the IAC (Indirect Access to Care) variable among the bootstrap replicates; on the graphs, an arrow points to the IAC if the frequency is ≥ 50%, from the IAC otherwise. IAMB = Interleaved Incremental Association.*

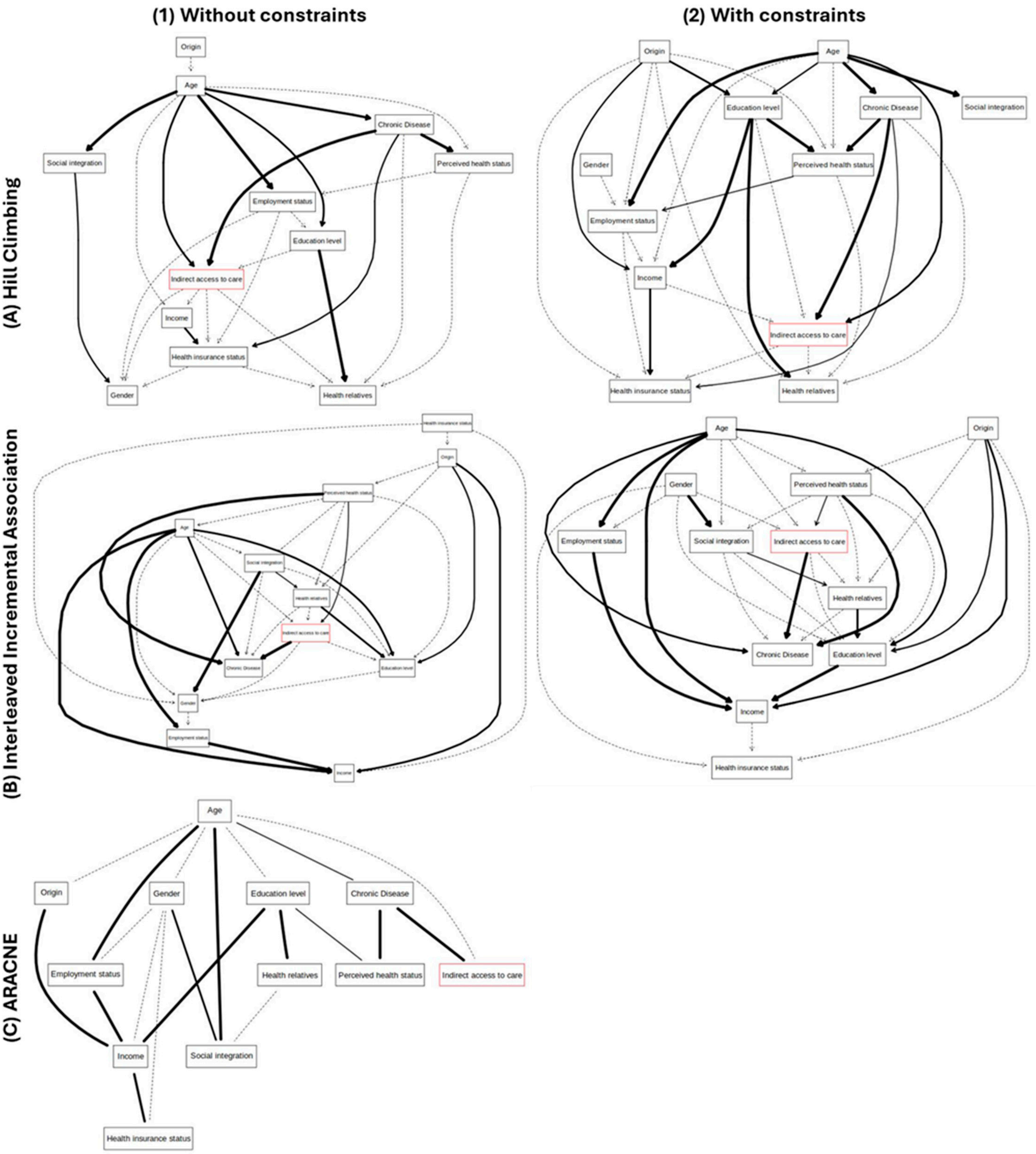

Figure S2. Final networks produced without (1) and with (2) knowledge constraints with Hill Climbing (A), Interleaved Incremental Association (B) and ARACNE (3) algorithms. The lines are plotted if the relative frequency of the links were ≥ 0.05 in the bootstrap replicates (n=1,000). When the relative frequency of the links in the bootstrap replicates were lower than 0.5, the lines are plotted as dashed lines. When the relative frequency of the links in the bootstrap replicates were higher than 0.5, the thickness of the line is proportional to the relative frequency.
